# Changes in adult smoking behaviours in ten global adult tobacco survey (GATS) countries during 2008–2018 - a test of ‘hardening’ hypothesis’

**DOI:** 10.1186/s12889-021-11201-0

**Published:** 2021-06-24

**Authors:** Chandrashekhar T Sreeramareddy, Saint Nway Aye

**Affiliations:** 1 Department of Community Medicine, International Medical University, Bukit Jalil, Kuala Lumpur, Malaysia; 2 Department of Pathology, International Medical University, Bukit Jalil, Kuala Lumpur, Malaysia

**Keywords:** Tobacco smoking behaviors, Hardcore smoking, Smoking Cessation, Nicotine dependence, Cross-Sectional Studies, Developing countries

## Abstract

**Background:**

Hardcore smoking behaviours and test of hardening are seldom reported from low-and-middle-income countries (LMICs). We report country-wise changes in smoking behaviors between two sequential surveys and explored ecologically the relationship between MPOWER scores and smoking behaviors including hardcore smoking.

**Methods:**

We analysed sequential Global Adult Tobacco Survey (GATS) data done at least at five years interval in 10 countries namely India, Bangladesh, China, Mexico, Philippines, Russia, Turkey, Ukraine, Uruguay, and Vietnam. We estimated weighted prevalence rates of smoking behaviors namely current smoking (both daily and non-daily), prevalence of hardcore smoking (HCS) among current smokers (HCSs%) and entire surveyed population (HCSp%), quit ratios (QR), and the number of cigarettes smoked per day (CPD). We calculated absolute and relative (%) change in rates between two surveys in each country. Using aggregate data, we correlated relative change in current smoking prevalence with relative change in HCSs% and HCSp% as well as explored the relationship of MPOWER score with relative change in smoking behaviors using Spearman’ rank correlation test.

**Results:**

Overall daily smoking has declined in all ten countries lead by a 23% decline in Russia. In India, Bangladesh, and Philippines HCSs% decreased as the smoking rate decreased while HCSs% increased in Turkey (66%), Vietnam (33%) and Ukraine (15%). In most countries, CPD ranged from 15 to 20 sticks except in Mexico (7.8), and India (10.4) where CPD declined by 18 and 22% respectively. MPOWER scores were moderately correlated with HCSs% in both sexes (*r* = 0.644, *p* = 0.044) and HCSp% (*r* = 0.632, *p* = 0.05) and among women only HCSs% (*r* = 0.804, *p* = 0.005) was significantly correlated with MPOWER score.

**Conclusion:**

With declining smoking prevalence, HCS had also decreased and quit rates improved. Ecologically, a positive linear relationship between changes in smoking and HCS is a possible evidence against ‘hardening’. Continued monitoring of the changes in quitting and hardcore smoking behaviours is required to plan cessation services.

## Background

Decreasing prevalence of tobacco smoking in many high-income countries leaves behind a subgroup of smokers who are unable to quit smoking as light smokers are more likely to quit than heavy (more dependent) smokers [1]. This has led to the concept of ‘hardening’, and that ‘hardened’ smokers would pose a greater challenge for further reduction of smoking prevalence [2]. Researchers have proposed that heavy smokers are more addicted and less likely to quit as they are unwilling or unable to quit smoking [3]. Hard-core smoking (HCS) was originally defined as those who have been smoking daily for a substantial period and are unable and/or unwilling to quit despite the knowledge about smoking hazards and faced with social disapprobation of their smoking behaviour [4]. Various studies from developed countries have variedly defined HCS using serial survey data on smoking behaviors and examined if the proportion of HCS among the smokers has increased over time as the prevalence of smoking declined [5]. However, evidence from developed countries on hardening is inconclusive since very few studies have reported that ‘hardening’ was occurring, [6–8] while recent studies have reported that ‘hardening’ was not occurring [9–11].

Lately even in low- and middle-income (LMICs) countries tobacco use is declining albeit at a slower rate [12]. Therefore, it would be interesting to examine if ‘hardening’ was occurring in developing countries as well. Thus far ‘hardening’ has been tested only in developed countries using national or sub national level serial survey data primarily aimed at monitoring time trends in tobacco use [13]. Nevertheless, such serial national survey data are unavailable for developing countries. To-date two studies have reported that varying proportions of HCS exist among smokers in LMICs [14, 15] but ‘hardening hypotheses has not yet been tested in developing countries. Rate of daily smoking, non-daily smoking, quit ratio, HCS, cigarettes smoked per day (CPD) were reported from Australia [13] to reflect the changes in tobacco use behaviours between 2001 and 2016. Sequential Global Adult Tobacco Surveys (GATS) data would be able to describe the changes in tobacco use behaviours rather than testing the hardening hypothesis. We estimated absolute and percentage (relative) changes in smoking behaviors (prevalence of daily, and non-daily smoking, quit ratio, cigarettes per day) and prevalence of HCS among current smokers (HCSs%) and the entire population (HCSp%) between two surveys done at an interval of at least five years in ten GATS countries. We also explored ecologically, if MPOWER score as a proxy indicator for tobacco control policy would explain the changes in the tobacco use behaviours and tested possible relationship between change in smoking rates and HCSs% and HCSp%.

## Methods

### Design

We included data from India, Bangladesh, China, Mexico, Philippines, Russia, Turkey, Ukraine, Uruguay, and Vietnam. Turkey has undergone three GATS surveys, in the years 2008, 2012 and 2016. We selected 2008 and 2016 for this analysis as the time difference was more than five years. Data from Thailand (2009 and 2011) which had undergone two rounds of surveys were not included since the interval between the surveys was less than five years and the most recent survey data was older than eight years.

### Data source

The Global Adult Tobacco Surveys (GATS) is a series of nationally representative, cross-sectional household surveys done as part of the Global Tobacco Surveillance System (GTSS) to monitor tobacco use. GATS data on tobacco use behaviours among civilian, non-institutionalised individuals aged 15 years and above are collected using a standardised questionnaire. The data is publicly available at (http://nccd.cdc.gov/gtssdata/Ancillary/DataReports.aspx?CAID=2). The eligible individuals were sampled using a stratified, multi-stage, probability sampling technique. In each sampled geographic location, the households were randomly selected and all eligible persons in each selected household were interviewed. However, only one household member was randomly selected and interviewed with a handheld device used for rostering and data collection. In each GATS country, a core GATS questionnaire was adapted to suit the local tobacco use context. Interviews were done privately by either a male or female interviewer in all the countries. However, in India, Bangladesh, Indonesia, and Qatar due to cultural sensitivity interviewers and respondents were of the same sex. Further details about the survey instrument, methodology etc. are published in detail elsewhere [16].

### Outcome variables

** 1) Daily smokers (DS):** Those who smoke at least one cigarette every day [17].

**2) Non-daily smokers (NDS):** Smokers who responded that they do not smoke every day [17].

**3) Current smokers:** Smokers who responded that they smoked either daily or non-daily (both DS and NDS).

**4) Hardcore smoking (HCS):** Consistent with our previous report based on GATS [15] the following five criteria were used to define HCS: 1) is a current daily smoker; 2) smokes 10 or more cigarettes per day; 3) smokes their first cigarette within 30 min after waking up; 4) has not made any quit attempts during the previous 12 months; and 5) has no intention to quit smoking at all or during the next 12 months.

**5) Cigarettes per day (CPD):** Total number of sticks (all types of smoking tobacco products) smoked each day was obtained by adding up the reported numbers for each type of smoking tobacco products.

**6) Prevalence of HCS among current smokers (HCSs%):** Percentage of current smokers classified as HCS among current smokers only.

**7) Prevalence of HCS in the entire survey population (HCSp%):** Percentage of current smokers classified as HCS among the entire survey population.

**8) Quit ratio:** The ratio of former (past) smokers to ever smokers. Ever smokers includes both current and former smokers, daily as well as non-daily [18]. Quit ratios (QR) were estimated using the raw number of smokers (both current and former smokers inclusive of daily and non-daily smokers).

### MPOWER score

To estimate the extent of tobacco control policy in each country, we created a composite score using MPOWER strategies of World Health Organisation. We extracted MPOWER data from WHO reports on the global tobacco epidemic [19]. MPOWER score provides a snapshot of tobacco control policy as reported in other studies that tested its association with smoking prevalence [20, 21]. For each of the measure, a score of 1 is ascertained if data was lacking or no recent data (since 2009) or data that is not both recent and representative of the national population, whereas scores 2 to 4 (for M) and 2–5 (for P, O, W, E and R) represent a scale from weakest to strongest level of tobacco control policy in that country (Table 1). A score was ascertained for each of the six dimensions of MPOWER and the total score was the sum of the scores for all six dimensions of MPOWER. Thus, the highest possible score for each country was 29.

**Table 1:** Components, their definition and score range of WHO’s MPOWER composite score

Component	Definition	Score range
**M**	Monitor tobacco use and prevention policies	1–4
**P**	Protect people from tobacco smoke	2–5
**O**	Offer help to quit tobacco use	2–5
**W**	Warn about the dangers of tobacco	2–5
**E**	Enforce bans on tobacco advertising, promotion, and sponsorship	2–5
**R**	Raise taxes on tobacco	2–5

### Statistical analysis

Weighted prevalence rates of DS, NDS, CS and HCS were estimated using Stata 11.2 for each country and survey. All estimates were computed for both sexes and overall, in each country and survey. In addition, proportion of all smokers who were defined HCS were estimated. To assess the change in smoking behaviours between two sequential surveys, we estimated both absolute and relative (percentage) differences in aggregate (at country-level) smoking behaviors defined as above. To test statistical significance of change in estimates between two surveys, we calculated Wald statistics (difference/estimated standard error) using aggregate data of each outcome i.e., prevalence estimates and 95% CIs for each survey. CPD used for correlation analyses was the average number of cigarettes smoked daily as a country-level aggregate using the total sticks of cigarettes (smoking tobacco).

**Fig. 1:**
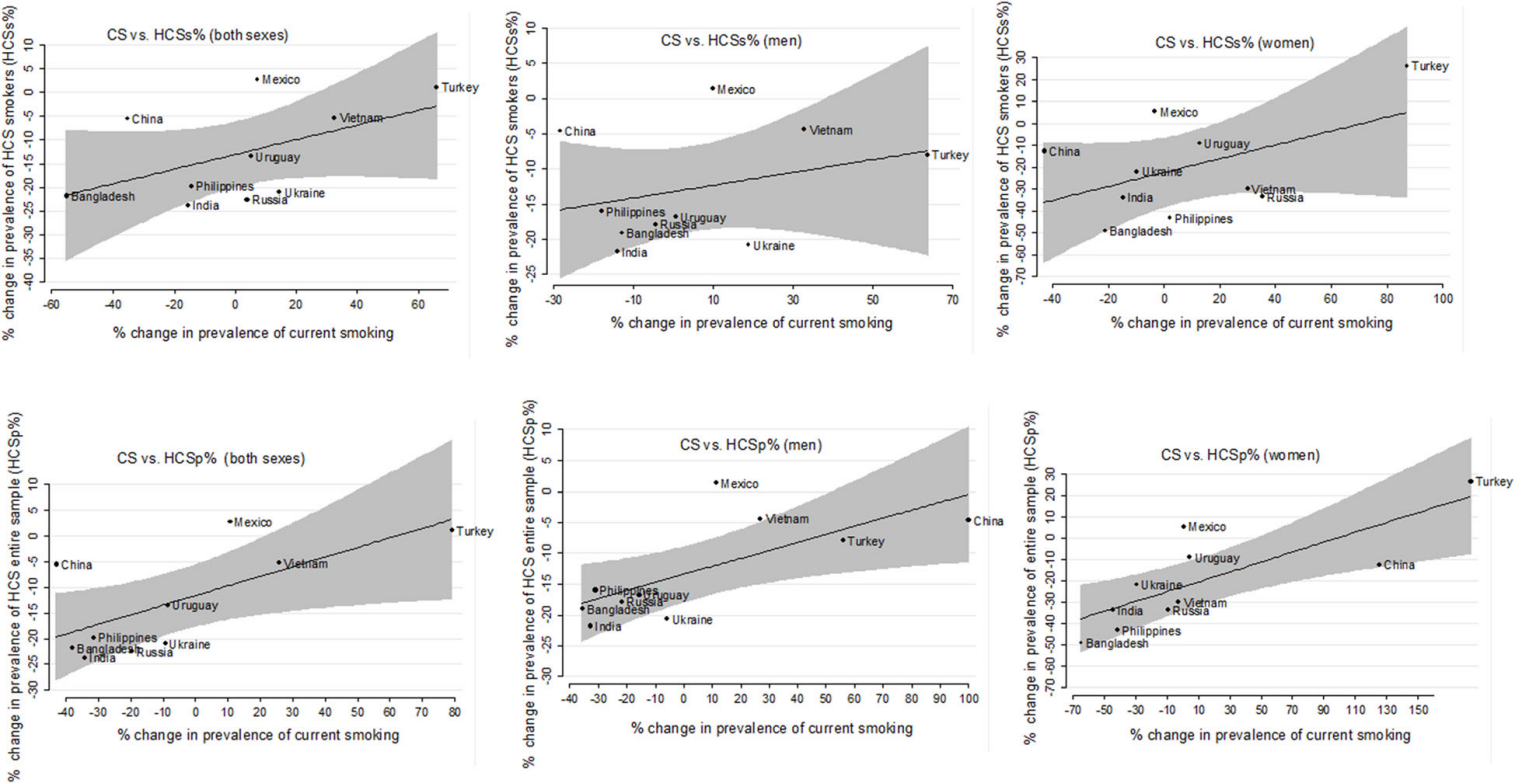
Two-way scatter plots of comparison changes in prevalence of CS with changes in HCSs% and HCSp% among men, women and both sexes

At an aggregate (country) level, we also explored the relationship between MPOWER scores and percentage change in each smoking behaviour. We also illustrated a possible association between change of prevalence of CS and change in HCSs% and HCSp% using Spearman’s rank correlation test as well as two-way scatter plots (Fig. 1). A *p*-value of < 0.05 was considered as statistically significant.

## Results

Tables 2, 3 and 4 show aggregate rates of smoking behaviours among both sexes, male and female along with absolute and percentage changes in 10 GATS countries. Changes in smoking behaviours and constructs of HCS and MPOWER scores for each country are shown in the appendix. Overall (both sexes), DS prevalence declined in eight of the 10 countries in absolute terms i.e., prevalence points ranging from 0.2 in Vietnam to 7.7 in Russia. However, the decline was statistically significant in all countries except China, Mexico, and Vietnam. Russia (22.9%), Ukraine (21.2%), Bangladesh (21.5%) and India (19.4%) were among the countries with highest percentage decline in overall DS. Notably, in Turkey, overall DS had increased by 2.2 points (8%) between 2008 and 2016 whereas in Mexico DS was nearly the same (sex-wise as well).

**Table 2:** Prevalence estimates of daily smoking, non-daily smoking, and current smoking their 95% CIs in ten countries; absolute difference and percentage change between baseline and most recent rounds of GATS

	Daily smoking			Non-daily smoking			Current smoking		
	men	women	both sexes	men	women	Both sexes	men	women	both sexes
India 2009	18.3 (17.3, 19.3)	2.4 (2.1,2.8)	10.7 (10.1, 11.2)	5.9 (5.4,6.4)	0.5 (0.4,0.6)	3.3 (10.1,11.2)	24.3 (23.3,25.3)	2.9 (2.5,3.4)	13.9 (13.4, 14.5)
India 2016	15.2 (14.5, 15.9)	1.7 (1.4,1.9)	8.6 (8.2,9.0)	3.8 (3.4,4.2)	0.3 (0.2,0.4)	2.1 (1.9, 2.3)	19.0 (18.1, 19.9)	1.9 (1.7, 2.2)	10.7 (10.2, 11.1)
**Absolute change**	**−3.1**	**− 0.8**	**−2.1**	**−2.1**	**− 0.2**	**−1.2**	**− 5.3**	**−1.0**	**−3.3**
**% change**	**−17.1**	**−31.9**	**− 19.4**	**− 36.0**	**− 41.2**	**− 37.0**	**− 21.7**	**−33.6**	**− 23.7**
***P*-value**	**< 0.001**	0.001	**< 0.001**	**< 0.001**	0.005	**< 0.001**	**< 0.001**	**< 0.001**	**< 0.001**
Bangladesh 2009	40.7 (38.5, 42.9)	1.3 (0.9, 1.8)	20.9 (19.8,22.0)	4.0 (3.2, 4.9)	0.2 (0.0, 0.3)	2.1 (1.7, 2.5)	44.7 (42.5, 46.9)	1.5 (1.0, 2.0)	23.0 (21.8, 24.1)
Bangladesh 2017	33.1 (31.2, 35.1)	0.7 (0.4, 0.9)	16.4 (15.5, 17.4)	3.1 (2.4, 3.8)	0.1 (0.0, 0.2)	1.5 (1.2, 1.9)	36.2 (34.2, 38.2)	0.8 (0.5, 0.1)	18.0 (17.0, 18.9)
**Absolute change**	**−7.6**	**−0.6**	**−4.5**	**− 0.9**	**− 0.1**	**−0.5**	**−8.5**	**− 0.7**	**−5.0**
**% change**	**− 18.6**	**− 43.6**	**− 21.5**	**− 23.1**	**−38.9**	**−26.2**	**−19.0**	**− 49.0**	**− 21.8**
***P*-value**	< 0.001	0.022	**< 0.001**	0.109	0.277	0.027	< 0.001	0.011	**< 0.001**
China 2010	45.3 (42.7, 48.0)	2.0 (1.5, 2.6)	24.1 (22.5, 25.6)	7.6 (6.5, 8.6)	0.4 (0.2, 0.6)	4.0 (3.5, 4.6)	52.9 (50.6, 55.2)	2.4 (1.8, 3.0)	28.1 (26.6, 29.6)
China 2018	44.4 (42.3, 46.4)	1.6 (1.2, 4.6)	23.2 (22.0, 24.5)	6.1 (5.4, 6.9)	0.5 (0.3, 0.6)	3.3 (2.9, 3.8)	50.5 (48.6, 52.4)	2.1 (1.6, 2.5)	26.6 (25.4, 27.8)
**Absolute change**	**−0.9**	**− 0.4**	**−0.8**	**−1.5**	**0.1**	**− 0.7**	**−2.4**	**− 0.3**	**− 1.5**
**% change**	**−2.1**	**− 20.0**	**−3.6**	**− 19.3**	**21.9**	**− 20.1**	**−4.5**	**− 12.5**	**−5.8**
***P*-value**	0.599	0.661	0.376	0.023	0.346	0.054	0.115	0.433	0.126
Mexico 2009	11.8 (10.6,13.0)	3.7 (2.9, 4.5)	7.5 (6.8, 8.3)	13.0 (11.8,14.2)	4.1 (3.3, 4.9)	8.4 (7.5, 9.2)	24.8 (23.1, 26.5)	7.8 (6.6, 9.0)	15.9 (14.7, 17.1)
Mexico 2015	11.9 (10.6,13.2)	3.6 (2.9, 4.3)	7.6 (6.8, 8.3)	13.3 (12.0, 14.5)	4.6 (3.9, 5.3)	8.8 (8.0, 9.5)	25.2 (23.5, 26.8)	8.2 (7.2, 9.2)	16.3 (15.4, 17.3)
**Absolute change**	**0.1**	**−0.0**	**0.0**	**0.3**	**0.5**	**0.4**	**0.3**	**0.4**	**0.4**
**% change**	**0.8**	**−1.1**	**0.5**	**2.1**	**12.2**	**4.9**	**1.4**	**5.6**	**2.8**
***P*-value**	0.867	0.853	0.750	0.626	0.121	0.23	0.612	0.35	0.361
Philippines2009	38.2 (36.3, 40.1)	6.9 (5.9, 7.8)	22.5 (21.4, 23.6)	9.4 (8.2, 10.6)	2.1 (1.5, 2.6)	5.7 (5.0, 6.4)	47.6 (45.7, 49.6)	9.0 (7.9, 10.0)	28.2 (27.0, 29.5)
Philippines 2015	33.9 (32.1, 35.7)	3.6 (3.0, 4.2)	18.7 (17.7, 19.7)	6.4 (5.5, 7.3)	1.5 (1.1,1.9)	3.9 (3.4, 4.5)	40.0 (38.5, 42.1)	5.1 (4.4, 5.8)	22.7 (21.6, 23.7)
**Absolute change**	**−4.4**	**−3.2**	**−3.8**	**−3.0**	**−0.6**	**−1.8**	**−7.6**	**−3.8**	**−5.6**
**% change**	**−11.4**	**−47.9**	**− 16.9**	**− 31.6**	**− 28.1**	**− 31.0**	**−16.0**	**− 43.0**	**− 19.8**
***P*-value**	0.001	**< 0.001**	**< 0.001**	**< 0.001**	0.084	**< 0.001**	**< 0.001**	**< 0.001**	**< 0.001**
Russia 2009	55.0 (53.1, 56.8)	16.3 (14.4,18.1)	33.8 (32.5, 35.1)	5.2 (4.5, 6.0)	5.4 (4.6, 6.3)	5.3 (4.7, 6.0)	60.2 (58.4, 62.0)	21.7 (19.6,23.8)	39.1 (37.8, 40.5)
Russia 2016	43.9 (41.9, 45.8)	11.3 (10.2,12.4)	26.0 (24.9, 27.2)	5.6 (4.7, 6.6)	3.1 (2.5, 3.7)	4.3 (3.7, 4.8)	49.5 (47.5, 51.5)	14.4 (13.0, 15.8)	30.3 (28.9, 31.7)
**Absolute change**	**−11.1**	**−5.0**	**−7.7**	**0.4**	**−2.3**	**−1.1**	**− 10.7**	**− 7.2**	**−8.8**
**% change**	**−20.2**	**−30.6**	**−22.9**	**7.2**	**−41.8**	**− 20.2**	**−17.8**	**−33.5**	**− 22.6**
***P*-value**	< 0.001	**< 0.001**	0.0007	0.356	**< 0.001**	0.021	**< 0.001**	**< 0.001**	**< 0.001**
Turkey 2008	43.8 (41.8, 45.9)	11.6 (10.4, 12.7)	27.4 (26.2, 28.6)	4.1 (3.3, 4.8)	3.6 (2.9, 4.3)	3.8 (3.3, 4.3)	47.9 (45.8, 50.0)	15.2 (13.9, 16.4)	31.2 (29.9, 32.5)
Turkey 2016	41.8 (39.9, 43.7)	17.5 (15.8, 19.2)	29.6 (28.2, 31.0)	2.3 (1.8, 2.8)	1.7 (1.2, 2.1)	2.0 (1.6, 2.4)	44.1 (42.2, 43.51)	19.2 (17.4, 20.9)	31.6 (30.2, 33.6)
**Absolute change**	**−2.0**	**5.9**	**2.2**	**−1.8**	**−1.9**	**−1.8**	**−3.8**	**4.0**	**0.4**
**% change**	**−4.6**	**50.8**	**8.0**	**−43.9**	**−52.9**	**−48.0**	**−7.9**	**26.3**	**1.1**
***P*-value**	0.161	**0.001**	0.019	**< 0.001**	**< 0.001**	**< 0.001**	0.001	< 0.001	0.565
Ukraine2010	45.5 (43.5, 47.4)	8.9 (7.6,10.1)	25.5 (24.4, 26.6)	4.5 (3.6, 5.5)	2.4 (1.7, 3.0)	3.4 (2.8, 3.9)	50.0 (48.1, 51.9)	11.2 (9.9, 12.6)	28.9 (27.7, 30.0)
Ukraine2017	35.9 (33.9, 37.8)	7.0 (5.8, 8.1)	20.1 (18.9, 21.2)	3.8 (2.9, 4.7)	1.8 (1.2, 2.4)	2.7 (2.1, 3.3)	39.7 (37.8, 41.6)	8.8 (7.5, 10.1)	22.8 (21.5, 24.0)
**Absolute change**	**−9.6**	**−1.9**	**−5.4**	**−0.7**	**−0.6**	**−1.7**	**− 10.4**	**−2.4**	**−6.1**
**% change**	**−21.2**	**− 21.5**	**− 21.2**	**−16.0**	**− 23.5**	**− 19.0**	**−20.7**	**− 21.8**	**− 21.0**
***P*-value**	**< 0.001**	0.028	**< 0.001**	0.294	0.187	0.092	**< 0.001**	0.012	**< 0.001**
Uruguay 2009	24.8 (22.4, 27.2)	16.4 (14.8, 18.1)	20.4 (19.1, 21.8)	5.8 (4.6, 7.1)	3.3 (2.5, 4.1)	4.5 (3.8, 5.3)	30.7 (28.1, 33.3)	19.8 (8.0, 21.5)	24.9 (23.3, 26.6)
Uruguay 2017	21.5 (19.5, 23.5)	15.3 (13.7, 16.8)	18.3 (17.6, 19.5)	4.0 (3.0, 5.0)	2.7 (2.0, 3.4)	3.3 (2.8, 3.9)	25.6 (23.4, 27.7)	18.0 (16.3, 19.7)	21.6 (20.3, 22.9)
**Absolute change**	**−3.3**	**−1.1**	**−2.2**	**−1.8**	**−0.6**	**− 1.2**	**−5.1**	**− 1.8**	**−3.3**
**% change**	**− 13.3**	**−6.9**	**− 10.6**	**− 31.3**	**− 18.8**	**− 0.3**	**−16.7**	**−8.9**	**− 13.4**
***P*-value**	0.038	0.341	0.013	0.028	0.269	0.011	0.003	0.612	0.002
Vietnam 2010	38.7 (36.9, 40.6)	1.2 (0.7, 1.7)	19.4 (18.4, 20.5)	8.7 (7.5, 9.8)	0.2 (0.1,0.4)	4.3 (3.8, 4.9)	47.4 (45.4, 49.4)	1.4 (0.9, 2.0)	23.8 (22.6,24.9)
Vietnam 2015	38.7 (36.6, 40.9)	0.9 (0.5, 1.2)	19.2 (18.0, 20.4)	6.6 (5.6, 7.6)	0.2 (0.1, 0.4)	3.3 (2.8,3.8)	45.3 (43.1, 47.5)	1.0 (0.7, 1.5)	22.5 (21.2, 23.8)
**Absolute change**	**0.0**	**−0.3**	**−0.2**	**−2.1**	**0**	**−1.0**	**−2.1**	**− 0.4**	**−1.2**
**% change**	**0.1**	**−28.1**	**−1.2**	**−0.24**	**0.0**	**−23.6**	**−4.4**	**−29.9**	**−5.3**
***P*-value**	1.0	0.335	0.806	0.007	0.853	0.009	0.166	0.248	0.142

**Table 3:** Estimates of quit ratio, mean number of cigarettes smoked per day (CPD) in ten countries; absolute difference and percentage change between baseline and most recent GATS

	Quit ratio			Number of cigarettes smoked per day (CPD)		
	Men	Women	Both sexes	Men	Women	Both sexes
India 2009	0.145	0.158	0.146	10.7 (10.2, 11.1)	8.2 (6.6, 9.8)	13.3 (12.2, 14.3)
India 2016	0.198	0.2	0.198	14.0 (12.8, 15.2)	6.2 (5.1, 7.2)	10.4 (9.9, 10.8)
**Absolute change**	**0.053**	**0.042**	**0.052**	**3.3**	**−2.0**	**−2.9**
**% change**	**36.6**	**26.6**	**35.6**	**31.4**	**−24.4**	**−21.7**
***P*-value**				**< 0.001**	**0.040**	**< 0.001**
Bangladesh 2009	0.1920	0.402	0.202	12.3 (11.8, 12.8)	7.9 (4.7, 11.1)	12.2 (11.6, 12.7)
Bangladesh 2017	0.2290	0.602	0.240	11.5 (10.8, 12.1)	7.6 (5.1, 10.2)	11.4 (10.7, 12.1)
**Absolute change**	**0.04**	**0.20**	**0.04**	**−0.8**	**−0.2**	**− 0.8**
**% change**	19.2	49.0	**18.8**	−6.5	−3.2	**−6.5**
***P*-value**				**0.056**	**0.886**	**0.078**
China 2010	0.172	0.230	0.176	16.3 (15.8, 16.9)	12.4 (11.2, 13.7)	16.2 (15.6, 16.8)
China 2018	0.233	0.331	0.239	18.3 (17.7, 19.0)	14.2 (12.3, 16.1)	18.2 (17.6, 18.8)
**Absolute change**	**0.061**	**0.10**	**0.06**	**2.0**	**1.7**	**2.0**
**% change**	35.4	43.9	26.3	12.2	14.0	11.0
*P***-value**				**< 0.001**	**0.12**	**< 0.001**
Mexico 2009	0.507	0.565	0.521	9.8 (8.6, 11.1)	8.4 (6.1, 10.6)	9.5 (8.2, 10.7)
Mexico 2015	0.511	0.641	0.554	8.1 (7.3, 8.9)	6.9 (5.9, 7.8)	7.8 (7.2, 8.4)
**Absolute change**	**0.004**	**0.076**	**0.033**	**−1.7**	**−1.5**	**−1.7**
**% change**	**0.8**	**13.5**	**6.3**	**− 17.6**	**− 17.6**	**− 17.6**
**P-value**				**0.025**	**0.228**	**0.016**
Philippines 2009	0.277	0.409	0.303	11.1 (10.6, 11.7)	6.7 (5.9, 7.6)	10.4 (9.9, 10.9)
Philippines 2015	0.247	0.379	0.268	11.2 (10.7, 11.8)	8.2 (6.5, 9.8)	10.9 (10.4, 11.5)
**Absolute change**	**−0.03**	**−0.03**	**−0.035**	**0.1**	**1.47**	**0.48**
**% change**	**−10.8**	**−7.3**	**−11.6**	**0.90**	**21.9**	**4.60**
**P-value**				**0.871**	**0.113**	**0.239**
Russia 2009	0.238	0.331	0.26	17.0 (16.4, 17.5)	10.9 (9.9, 11.8)	15.4 (14.8, 15.9)
Russia 2016	0.3	0.659	0.335	17.0 (16.4, 17.7)	14.4 (12.9, 15.9)	16.4 (15.7, 17.0)
**Absolute change**	**0.062**	**3.28**	**0.075**	**0.1**	**3.5**	**1.0**
**% change**	**26.1**	**99.1**	**28.9**	**0.4**	**32.4**	**6.8**
**P-value**				**1.00**	**< 0.001**	**0.021**
Turkey 2008	0.356	0.415	0.371	19.4 (18.8, 20.0)	12.1 (11.2, 13.1)	17.8 (17.2, 18.4)
Turkey 2016	0.182	0.165	0.177	19.2 (18.3, 20.2)	20.2 (14.7, 25.8)	19.5 (17.7, 21.4)
**Absolute change**	**−0.174**	**−0.25**	**− 0.194**	**− 0.2**	**8.1**	**1.7**
**% change**	**−48.9**	**−60.2**	**−52.3**	**−0.9**	**66.5**	**9.4**
**P-value**				**0.727**	**0.005**	**0.087**
Ukraine 2010	0.335	0.449	0.355	17.2 (16.1, 17.8)	11.1 (10.1, 12.0)	16.0 (15.5, 16.5)
Ukraine 2017	0.402	0.454	0.412	18.2 (17.7, 18.7)	12.6 (11.7, 1 3.5)	17.2 (16.7, 17.6)
**Absolute change**	**0.067**	**0.005**	**0.057**	**1.0**	**1.5**	**1.1**
**% change**	**20.0**	**1.1**	**16.1**	**6.1**	**13.9**	**7.1**
**P-value**				**0.246**	**0.032**	**0.0005**
Uruguay 2009	0.497	0.488	0.493	16.8 (15.5, 18.1)	12.5 (11.4, 13.5)	15.0 (14.0, 15.9)
Uruguay 2017	0.571	0.549	0.561	16.7 (15.5, 17.9)	13.3 (12.3,14.3)	15.2 (14.4, 16.0)
**Absolute change**	**0.074**	**0.061**	**0.068**	**−0.1**	**0.8**	**0.2**
**% change**	**14.9**	**12.5**	**13.8**	**−0.6**	**6.5**	**1.5**
**P-value**				**0.912**	**.0.387**	**0.775**
Vietnam 2010	0.305	0.44	0.311	14.6 (13.7, 15.4)	10.0 (8.1, 11.9)	14.4 (13.6, 15.2)
Vietnam 2015	0.326	0.448	0.331	15.2 (14.6, 15.7)	11.5 (8.8, 14.2)	15.1 (14.4, 15.6)
**Absolute change**	**0.021**	**0.008**	**0.02**	**0.6**	**1.5**	**0.6**
**% change**	**6.9**	**1.8**	**6.4**	**4.0**	**15.2**	**4.5**
**P-value**				**0.442**	**0.027**	**0.175**

**Table 4:** Prevalence estimates of HCS among current smokers and entire survey population, their 95% CIs in ten countries; absolute difference and percentage change between baseline and most recent GATS

	HCS among current smokers (HCSs%)			HCS in the entire survey population (HCSp%)		
	Men	Women	Both sexes	Men	Women	Both sexes
India 2009	18.4 (16.8, 20.0)	10.4 (7.2, 13.5)	18.0 (16.1, 19.0)	4.5 (4.0, 4.9)	0.3 (0.2, 0.4)	2.5 (2.2, 2.7)
India 2016	15.8 (14.3, 17.3)	8.8 (5.4, 12.2)	15.2 (13.8, 16.5)	3.0 (2.7, 3.28)	0.2 (0.1, 0.2)	1.6 (1.5, 1.8)
**Absolute change**	**−2.6**	**−1.6**	**− 2.8**	**−1.46**	**−0.2**	**− 0.8**
**% change**	**−14.3**	**− 15.0**	**− 15.6**	**−32.7**	**− 45.2**	**−34.2**
P-value	0.020	0.50	0.006	< 0.001	0.079	< 0.001
Bangladesh 2009	16.7 (14.1, 19.3)	15.0 (1.5, 28.5)	32.2 (31.1, 33.3)	7.5 (6.2, 8.7)	0.2 (0.1, 0.5)	3.8 (3.4, 4.3)
Bangladesh 2017	14.5 (11.9, 17.0)	11.8 (1.3, 22.2)	14.4 (11.9, 16.9)	4.8 (3.9, 5.6)	0.1 (0.1, 0.2)	2.4 (2.0, 2.8)
**Absolute change**	**−2.2**	**−3.2**	**−17.8**	**− 2.7**	**−0.2**	**− 1.5**
**% change**	**− 13.1**	**−21.3**	**−55.2**	**−35.8**	**− 65.2**	**− 37.9**
P-value	0.242	0.713	< 0.001	0.001	0.153	< 0.001
China 2010	32.3 (31.1, 33.3)	30.9 (27.1, 34.8)	35.5 (34.5, 36.5)	5.1 (4.4, 5.9)	0.1 (0.1, 0.2)	2.7 (2.3, 3.0)
China 2018	23.1 (20.1, 25.4)	17.6 (10.5, 24.7)	22.9 (20.6, 25.1)	10.2 (9.1,11.3)	0.3 (0.1, 0.4)	5.3 (4.7, 5.9)
**Absolute change**	**−9.2**	**−13.3**	**−12.6**	**5.1**	**0.2**	**4.0**
**% change**	**−28.5**	**−43.0**	**−55.1**	**100**	**125**	**42.9**
P-value	< 0.001	0.001	< 0.001	< 0.001	< 0.001	< 0.001
Mexico 2009	4.6 (3.1, 6.1)	2.8 (0.6, 4.9)	4.1 (2.9, 5.4)	1.2 (0.8, 1.5)	0.2 (0.1, 0.4)	0.7 (0.45, 0.87)
Mexico 2015	5.1 (3.4, 6.76)	2.7 (1.1, 4.2)	4.4 (3.2, 5.7)	1.3 (0.8, 1.7)	0.2 (0.01–0.3)	0.7 (0.51, 0.94)
**Absolute change**	**0.5**	**−0.1**	**0.3**	**0.13**	**0**	**0.1**
**% change**	**9.7**	**−3.3**	**7.2**	**11.3**	**0**	**10.6**
P-value	0.663	0.941	0.739	0.621	1.0	1.0
Philippines 2009	22.0 (19.7, 24.4)	8.2 (5.0, 11.4)	19.8 (17.7, 21.9)	10.6 (9.4, 11.8)	0.7 (0.4, 1.0)	5.6 (4.9, 6.3)
Philippines 2015	18.0 (15.8, 20.2)	8.4 (4.6, 12.1)	16.9 (14.9, 19.0)	7.3 (6.3, 8.2)	0.4 (0.2, 0.6)	3.8 (3.3, 4.4)
**Absolute change**	**−4.0**	**0.2**	**−2.9**	**−3.3**	**−0.3**	**−1.8**
**% change**	**−18.2**	**2.0**	**−14.5**	**−31.2**	**− 41.9**	**− 31.4**
P-value	0.015	0.707	0.052	< 0.001	0.14	< 0.001
Russia 2009	39.5 (36.9, 42.1)	18.4 (15.1, 21.8)	33.1 (30.9, 35.3)	23.8 (22.2, 25.4)	4.0 (3.2, 4.8)	13.0 (12.0, 13.9)
Russia 2016	37.6 (34.8, 40.5)	24.9 (20.9, 29.0)	34.3 (31.7, 36.9)	18.6 (17.0, 20.3)	3.6 (2.9, 4.3)	10.4 (9.5, 11.4)
**Absolute change**	**−1.9**	**6.5**	**1.2**	**−5.2**	**−0.4**	**−2.6**
**% change**	**−4.7**	**35.3**	**3.7**	**−21.7**	**−10**	**− 19.7**
*P*-value	0.334	0.015	0.489	< 0.001	0.46	0.001
Turkey 2008	21.0 (18.7, 23.4)	16.1 (11.9, 20.2)	20.0 (17.8, 22.1)	9.2 (8.1, 10.4)	1.9 (1.3, 2.4)	5.5 (4.8, 6.1)
Turkey 2016	34.4 (31.5, 37.3)	30.1 (25.6, 34.5)	33.1 (30.5, 30.8)	14.4 (13.0, 15.8)	5.3 (4.4, 6.2)	9.8 (8.9, 10.1)
**Absolute change**	**13.4**	**14.0**	**13.2**	**5.6**	**3.4**	**4.3**
**% change**	**63.6**	**87.1**	**66.0**	**55.9**	**183.3**	**79**
P-value	< 0.001	< 0.001	< 0.001	< 0.001	< 0.001	< 0.001
Ukraine 2010	33.3 (30.8, 35.8)	21.2 (15.7, 26.7)	30.7 (28.5, 32.9)	16.7 (15.2, 18.1)	2.4 (1.7, 3.1)	8.9 (8.1, 9.6)
Ukraine 2017	39.5 (36.2, 42.8)	19.2 (13.5, 24.8)	35.2 (32.3, 38.1)	15.7 (14.1, 17.7)	1.7 (1.1, 2.3)	8.0 (7.3, 8.8)
**Absolute change**	**6.2**	**−2.1**	**4.5**	**−1**	**−0.7**	**−0.8**
**% change**	**18.7**	**−9.8**	**14.7**	**−5.9**	**−30.1**	**−9.5**
P-value	0.003	0.619	0.015	0.396	0.614	0.096
Uruguay 2009	17.6 (13.5, 21.7)	14.1 (9.6, 18.4)	16.1 (13.0, 19.1)	4.4 (3.2, 5.5)	2.3 (1.5, 3.1)	3.3 (2.6, 3.9)
Uruguay 2017	17.7 (13.6, 21.7)	15.9 (12.4, 19.4)	16.9 (13.9, 19.8)	3.7 (2.8, 4.5)	2.4 (1.8, 3.0)	3.0 (2.5, 3.5)
**Absolute change**	**0.1**	**1.8**	**0.8**	**−0.7**	**0.1**	**− 0.3**
**% change**	**0.5**	**12.6**	**5.0**	**−15.8**	**3.9**	**−8.5**
P-value	0.928	0.056	0.289	0.337	0.803	0.473
Vietnam 2010	20.8 (18.8, 22.9)	21.8 (14.0, 29.6)	20.9 (18.8, 22.9)	9.9 (8.8, 11.0)	0.3 (0.2, 0.5)	4.9 (4.4, 5.5)
Vietnam 2015	27.7 (25.3, 30.0)	28.3 (14.0, 42.6)	27.7 (25.4, 30.0)	12.5 (11.3, 13.8)	0.3 (0.1, 0.5)	6.2 (5.6, 6.9)
**Absolute change**	**6.8**	**6.5**	**6.8**	**2.7**	**−0.1**	**1.3**
**% change**	**32.7**	**29.8**	**32.6**	**26.9**	**−3.1**	**25.8**
P-value	< 0.001	0.434	0 < 0.001	0.002	0.937	0.002

Sex-wise, DS prevalence among women was lower than men and DS prevalence declines were seen among men as well as women in most countries except for the around 50% increase among Turkish women an increase by 5.9 prevalence points i.e., from 11.6 to 17.5. However, the sex-wise change in prevalence of DS was not statistically significant in China, Mexico, and Vietnam (both men and women), Turkey (men only) and Uruguay (women only). In Bangladesh (43.6 vs. 18.6), Philippines (47.9 vs. 11.4), India (32.0 vs.17.1) and Russia (30.6 vs. 20.2) the percentage decline in DS among women was much higher than among men (Table 2).

NDS prevalence was generally much lower than DS prevalence at both baseline and most recent surveys in all countries except Mexico. In Mexico, NDS prevalence was slightly higher than DS overall as well as sex-wise. In nine countries (excluding Mexico), overall NDS prevalence was < 6 and NDS prevalence had declined. Among the countries where NDS significantly declined Turkey (48%), India (37%) and Philippines (31%) had experienced large percentage declines. Notably in Mexico, NDS had increased by 5% overall as well as sex-wise. In the remaining nine countries, NDS prevalence had declined among both men and women (except Russian men) However, in Mexico, and Ukraine, (both sexes), Bangladesh and Russia (men only), and Philippines (women only) change in NDS prevalence was not statistically significant. (Table 2).

Overall (both sexes) current smoking (CS) prevalence had declined in eight countries except in Mexico, and Turkey where current smoking increased by 2.8 and 1.1% respectively. Leading percentage decline in CS occurred in India (23.7%), Russia (22.6%), Bangladesh (21.8%), Ukraine (21.0%), and Philippines (19.8%). Overall CS had slightly increased in Mexico and Turkey. CS had declined in both sexes as well in all countries where prevalence of DS had declined. In Mexico CS slightly increased among men, while in Turkey, CS decreased among men and DS rates had increased among women (Table 2). The change in prevalence of CS was not statistically significant in China, Mexico, Vietnam (both sexes) and Uruguay (women only) (Table 2).

Overall quit ratio (QR) had decreased in Turkey and Philippines by 52.3 and 11.6% respectively. In these two countries, QR decreased sex-wise as well. Among the other countries, India (35.6%) and China (26.3%) lead the percentage increase in QR followed by Russia (28.9%). Notably, QR among Russian women had nearly doubled, from 0.331 to 0.659 an increase of 99% whereas in Mexico and Vietnam QR changed very little (only 6% increase). Despite the modest percentage increase in QR during two sequential surveys, QR in Mexico (0.55) and Uruguay (0.56) was much higher than India (0.20) and China (0.24) (Table 3).

Overall (both sexes) cigarettes per day (CPD) decreased by about 22% in India and 18% in Mexico. However, in other countries CPD had marginally increased ranging from 1.5% in Uruguay to 9.4% in Turkey and 12.4% in China. Remarkably, CPD rose by 67% in Turkish and 32% in Russian women. In most recent surveys, overall CPD was lowest in Mexico (7.8) followed by India (10.4), Philippines (10.9) and Bangladesh (11.4), while in other countries CPD ranged from 15 to 20. CPD was generally lower among women than men but among Turkish women CPD was 20.2 higher than men (19.2) (Table 3).

In four countries namely Bangladesh, China, Russia, and Ukraine about a third of all smokers were hardcore smokers (HCS) at the baseline. However, the highest percentage decline in prevalence of HCS among smokers (HCSs%) was seen in only Bangladesh (55.2%) and China (55.1%) while in Russia HCSs% remained nearly the same; in Ukraine HCSs% increased by 14.7%. In all countries except Mexico, Philippines, Uruguay and Vietnam, the change in HCSs% was statistically significant. In Bangladesh and China, despite low QR at the baseline survey, the QR had increased by 18.8 and 26.3% respectively. In India and Philippines, HCSs% was about 20% and in both countries HCSs% decreased by 15%. However, there was a significant increase of 66% in HCSs% from 20 to 33.1% in Turkey and 32.6% increase in Vietnam (20.9 to 27.7%). In Mexico, the HCSs% was rather low at 4.0% and nearly remained the same (Table 4).

Sex-wise the declines in HCSs% was much higher among women than men in both Bangladesh and China, while in Turkey and Vietnam HCSs% increase was marginally higher among women than men. However, the change in HCSs% was statistically significant in only China, Russia, and Turkey for women. (Table 3). Overall prevalence of HCSp% in the entire surveyed population was higher among men than women in most countries. Overall prevalence of HCSp% among men in both surveys was higher than 15 in Russia and Ukraine and notably in both these countries the prevalence had decreased significantly in Russia but not in Ukraine. In all other countries HCSp% decreased and percentage decline was generally higher among women than men (Table 3). HCSp% in both sexes significantly declined in India, Bangladesh, and Philippines. However, among men in Turkey and Vietnam the HCSp% significantly increased by 56% (9.2 to 14.4) and 27% (9.9 to 12.5) respectively. Notably in turkey HCSp% significantly increased among women as well (Table 4).

The prevalence of HCS in the entire surveyed population (HCSp%) varied across the 10 countries and also according to sex in the first surveys. Overall, HCSp% was higher in Russia (13.0), and Ukraine (8.9) than in other countries; however, sex-wise more countries namely Russia (23.8), Ukraine (16.7), Philippines (10.6), Vietnam (9.9) and Turkey (9.2) had high HCSp% among men than women. Overall, HCSp% decreased (8.5 to 42%) significantly except Ukraine and Uruguay. However, HCSp% increased significantly in Turkey (79%), China (43%) and Vietnam (25.8%) (Table 4).

In all surveys in 10 countries bivariate comparisons between prevalence of current smoking and prevalence of HCS entire population (HCSp%) showed moderate to strong positive correlation in both sexes (*r* = 0.853, *p* < 0.001), among men (*r* = 0.752, *p* < 0.001) and women (*r* = 0.816, *p* < 0.001). However, correlation between prevalence of current smoking and prevalence of HCS among current smokers (HCSs%) showed a moderate and significant positive correlation in both sexes (*r* = 0.677, *p* = 0.0011) and men (*r* = 0.712, *p* = 0.0004) but not among women (*r* = 0.070, *p* = 0.772) (data not shown).

Correlation between the relative change in prevalence of current smoking and prevalence of HCS in the entire sample (HCSp%) showed there was a positive and strong linear correlation (*r* = 0.62 & *p* = 0.053, *r* = 0.709 & *p* = 0.021, *r* = 0.903 & *p* = 0.003) among both sexes, men as well as women, respectively. Similarly, comparison of percentage change in current smoking and prevalence of HCS among current smokers (HCSs%) was weakly correlated in both sexes as well as men and women (*r* = 0.0.31–0.50, *p* > 0.05). In most countries barring some exceptions namely Mexico, Turkey, and Vietnam, both HCSs% and HCSp% decreased with decline in smoking prevalence (Fig. 1).

Table 5 shows that among the seven tobacco smoking indicators DS, NDS, CS, QR and CPD were not significantly correlated with MPOWER scores among either sexes or both sexes combined. Most of the correlations were either weak or there was no correlation. Among women HCSs% (*r* = 0.804, *p* = 0.005) was moderately and significantly correlated with MPOWER score. QR was negatively correlated among women (*r* = − 0.60, *p* = 0.056) as well as men (*r* = − 0.472, *p* = 0.168) with MPOWER scores but was not statistically significant. Among both sexes combined only HCSs% was moderately correlated with MPOWER score but was not statistically significant (*r* = 0.632, *p* = 0.05) whereas HCSp% was moderately correlated and was statistically significant (*r* = 0.644, *p* = 0.044).

**Table 5:** Spearman’s rank correlation test between MPOWER score and percentage change in tobacco smoking behaviours in 10 GATS countries (aggregate data)

	Men		Women		Both sexes	
	coefficient	p-value	coefficient	p-value	coefficient	p-value
**Daily smoking**	−0.239	0.506	0.387	0.270	0.104	0.774
**Non-daily smoking**	−0.374	0.287	−0.509	0.133	−0.534	0.112
**Current smoking**	−0.129	0.723	0.325	0.359	0.068	0.853
**Quit ratio**	− 0.472	0.168	− 0.620	0.056	−0.571	0.085
**Prevalence of HCS among current smokers (HCSs%)**	0.534	0.112	0.804	0.005	0.644	0.044
**Cigarettes per day**	−0.153	0.672	0.546	0.103	0.264	0.461
**Prevalence of HCS entire sample (HCSp%)**	0.055	0.880	0.288	0.419	0.632	0.050

## Discussion

We studied the changes in smoking behaviours using data from two sequential surveys in 10 GATS countries where the hardening hypothesis has not yet been tested due to the lack of serial cross-sectional survey data. In Turkey and Vietnam, HCSs% had increased with a marginal decrease in smoking rates between two surveys suggestive of ‘hardening’. With this exception, ecologically the change in smoking prevalence and change in HCS between two surveys was positively correlated. A positive correlation of smoking rates with HCS in all 10 GATS countries is also an evidence against “hardening”. MPOWER scores were also significantly correlated with changes in HCS specifically among women. Our results are supported by studies based on survey data in European countries [22, 23] and serial survey data in Australia [13].

In most countries smoking prevalence had decreased while QR had increased in eight of the 10 countries. However, CPD decreased in three countries only while it increased in other countries. In Bangladesh, Philippines, and India DS, NDS as well as HCSs% had declined. Mexico fared the best among the 10 countries with the lowest DS, QR, HCSs%, as well as CPD but had the highest NDS prevalence (8.8%). In Turkey, the indicators worsened between 2008 and 2016, as the overall DS, CPD and HCSs% increased which was mainly due to increased rates among Turkish women.

The finding of a decline in DS prevalence is supported by previous reports based on GATS and most countries are on track towards the target of 30% reduction in tobacco use by 2030 [24]. However, the authors presented prevalence of overall tobacco use only rather than detailed smoking behaviours which would help better understand the drivers of change in prevalence of tobacco use. Declining prevalence of DS and NDS may be attributed to the commitment shown by governments in these countries by ratification of Framework Convention on Tobacco Control [25] and rolling out evidence informed MPOWER tobacco control policies [26]. Contrary to ‘hardening hypothesis’, our analyses suggests that in two countries where DS prevalence had declined, HCSs% also decreased while NDS prevalence had decreased in 9/10 countries. Increasing QR in 8/10 countries supports our finding of decreased prevalence of DS and NDS. However, since GATS countries do not have serial surveys about smoking, to test ‘hardening’, we only assessed change in tobacco use behaviours between two sequential surveys. Though not a robust method to test ‘hardening’ hypothesis our analyses at least revealed that ‘hardening’ is unlikely to be occurring in the 10 GATS countries we analysed. An analysis of serial surveys in Victoria, Australia between 2011 and 2016 has showed that not only smoking prevalence but even the indicators of hardening had declined over the same period [13]. Ecologically, a positive correlation between relative changes in CS and HCSs% and HCSp% further provides weak evidence against ‘hardening.’ Although statistically not significant among men, correlation was moderate among both sexes. Our results imply that any decline in smoking prevalence would also result in a decrease in HCS rather than an increase in HCS as proposed by ‘hardening theory’.

How the ten GATS countries are faring towards achieving the WHO NCD target is interesting as the smoking patterns had changed favourably in a few countries only. Globally, tobacco control interventions are guided by the World Health Organization’s (WHO) Framework Convention on Tobacco Control (FCTC) and all these countries are signatories of the FCTC and are thus committed to a strong tobacco control policy [27]. The MPOWER interventions are at least expected to reduce the CPD, if not reduce the prevalence of smoking. However, our results are discouraging since CPD had decreased in three countries only i.e., India, Bangladesh, and Mexico. Despite countries having rolled out pictorial warnings on cigarette packs in addition to other interventions [28] CPD was higher than 15 in Russia, Ukraine, Turkey, Uruguay, China, and Vietnam. Mexico being one of the first countries to ratify FCTC, has shown very strong commitment to tobacco control policy implementation hence the decrease in DS prevalence [29] Nevertheless, NDS has increased in Mexico, perhaps as result of recreational smoking or daily smokers who have cut down on CPD or changed to non-daily smoking behaviour. India too has implemented tobacco control policies mostly centred around smoke-free policies but is lacking in cessation assistance and product regulations. We note that even though prevalence of DS and NDS marginally declined, QR was still very low despite its improvement (35% increase). An exception to the decline in smoking prevalence was Turkey where DS, HCSs%, CPD had increased overall after 2012. QR had fallen by about 50% and these changes were driven mainly by increasing smoking prevalence in mostly young women. Turkey indeed was hailed for its success in bringing down smoking prevalence in 2012 by its strong smoke-free policies and increased taxation [30]. A steep increase in CPD among women, increased HCSs% and decreased NDS prevalence and QR from 2012 suggest that the existing pool of smokers may have perhaps “hardened” coinciding with increased DS. As ‘hardening’ is related to CPD those who smoked more sticks would have higher dependence.

In Mexico, the pattern of change in smoking behaviour was in contrast to what has occurred in Turkey. Despite the stagnation of DS prevalence, NDS prevalence was higher than DS. In Mexico, QR was highest among the ten countries and had also increased, while CPD was low and had decreased. MPOWER scores of 29 in Turkey and 20 in Mexico are contradictory to these changes and suggests that Mexico had better implementation and enforcement of MPOWER strategies. MPOWER scores moderately correlated with percentage change in NDS and CPD though it was not statistically significant. India too had very remarkable decrease in not just smoking but all forms of tobacco use as we found that HCSs% and CPD had decreased as well. Positive correlation of MPOWER with HCS however, suggested that tobacco control policies possibly had an impact on reducing dependence. The changes were very much attributable to the tobacco control policies implemented in the country for over a decade [31]. Our results support the empirical evidence on effect of MPOWER on reduction of smoking prevalence [20, 21].

Our results have suggested that hardening does not seem to be occurring in all ten countries even though, we could not test ‘hardening hypothesis’ due to non-availability of serial survey data. Only in Turkey and Vietnam, HCSs% had increased with decreased DS prevalence. Our analyses revealed some important changes in smoking behaviours such as change in QR, and CPD indicating the impact of MPOWER strategies. In Mexico, the persistence of NDS is not only indicative of the success of tobacco control policies but also highlights the need for a changed approach to target NDS for utilisation of cessation services. Indicators such as HCSs% and CPD are suggestive of the level of dependence among the current smokers and are potentially helpful in planning cessation services. Thus, GATS survey is an important source of data for monitoring tobacco use behaviours and it is critical to implement GATS in more LMICSs at regular intervals to monitor progress towards health-related target of SDG (one third premature mortality from non-communicable diseases) [32] as well as WHO target (30% decline in prevalence of tobacco use) to be achieved by 2030 [33]. Changes in smoking behaviours showed heterogenous patterns in terms of tobacco use, and quitting behaviours across the countries and sex-wise.

Our findings must be carefully interpreted in the light of inherent limitations of design and availability of survey data. Self-reports about smoking behaviours are subject to reporting, recall and social desirability bias as detailed in our previous reports on GATS data [15]. Further information about number of cigarettes is likely to be under reported and inaccurate as smokers tend to round off to five,10 etc. (known as ‘heaping’) and do not report exact number of sticks smoked during the previous 24 h [34]. We examined a change in ten countries having data from at least five years intervals and the most recent survey done was during 2015 or later. Nevertheless, the implementation of tobacco control measures takes a longer time to bring about desired change; thus, attributing the changes to the tobacco control policies is difficult [35]. The MPOWER score we used in exploratory analyses does not measure actual enforcement and our ecological analyses lacked statistical power as only ten countries were analysed.

## Conclusion

A positive and strong correlation between decline in prevalence of smoking and hardcore smoking. Suggests that hardening does not seem to be occurring. Tobacco control strategies seems to have reduced not only smoking prevalence but also hardcore smoking particularly among women. Continued monitoring of hardcore smoking behaviours is needed to inform appropriate strategies for improved tobacco control, and achievement of the SDG and WHO target by 2030 as a measure of success of MPOWER strategies.

## Data Availability

The datasets generated and/or analysed during the current study are available in the Global Tobacco Surveillance System (GTSS) repository. https://www.cdc.gov/tobacco/global/gtss/gtssdata/index.html

## Abbreviations

DS: Daily smoker
NDS: Non-daily smoker
CS: Current smoker
HCS: Hardcore smoker
HCSs%: Prevalence of HCS among current smokers
HCSp%: Prevalence of HCS in overall population
QR: Quit ratio
